# Tsunami detection by GPS-derived ionospheric total electron content

**DOI:** 10.1038/s41598-021-92479-3

**Published:** 2021-06-21

**Authors:** Mahesh N. Shrivastava, Ajeet K. Maurya, Gabriel Gonzalez, Poikayil S. Sunil, Juan Gonzalez, Pablo Salazar, Rafael Aranguiz

**Affiliations:** 1 Department of Geological Sciences, Universidad Catolica del Norte, Antofagasta, Chile; 2 National Research Center for Integrated Natural Disaster Management, Santiago, Chile; 3 Department of Physics, Doon University, Dehradun, India; 4 Department of Marine Geology and Geophysics, School of Marine Sciences, Cochin University of Science and Technology, Kochi, India; 5 Department of Civil Engineering, Universidad Catolica de la Santísima Concepcion, Concepcion, Chile

**Keywords:** Natural hazards, Geophysics

## Abstract

To unravel the relationship between earthquake and tsunami using ionospheric total electron content (TEC) changes, we analyzed two Chilean tsunamigenic subduction earthquakes: the 2014 Pisagua M_w_ 8.1 and the 2015 Illapel M_w_ 8.3. During the Pisagua earthquake, the TEC changes were detected at the GPS sites located to the north and south of the earthquake epicenter, whereas during the Illapel earthquake, we registered the changes only in the northward direction. Tide-gauge sites mimicked the propagation direction of tsunami waves similar to the TEC change pattern during both earthquakes. The TEC changes were represented by three signals. The initial weaker signal correlated well with Acoustic Rayleigh wave (AW_Rayleigh_), while the following stronger perturbation was interpreted to be caused by Acoustic Gravity wave (AGW_epi_) and Internal Gravity wave (IGW_tsuna_) induced by earthquakes and subsequent tsunamis respectively. Inevitably, TEC changes can be utilized to evaluate earthquake occurrence and tsunami propagation within a framework of multi-parameter early warning systems.

## Introduction

Modern geodesy has improved significantly with the extensive use of Global Positioning System (GPS) techniques, allowing the detection of secular variations in the Earth’s surface position with unprecedented accuracy^1^. Modern seismology widely uses GPS to understand the nucleation and propagation of earthquake events. It has also been demonstrated that surface deformation related to earthquake and tsunami propagation produce ionospheric changes in the total electron content (TEC)^2–8^. As per the current knowledge of the seismic-ionospheric phenomena studied by many earlier researchers^8–14^ suggest observations of three waves, namely acoustic gravity waves at epicenter (AGW_epi_), internal gravity waves induced by Tsunami (IGW_tsuna_), and acoustic wave due to seismic Rayleigh wave (AW_Rayleigh_), following the nomenclature introduced by Occhipinti et al.^11^. We remind that AGW_epi_: in essence the acoustic-gravity wave generated at the epicentral area associated to the vertical displacement of the ground/ocean. It contains a more high‐frequency component (0.5–1 Hz). These waves propagate with speed of around 500–1000 m/s and reach the ionosphere ~ 8 min after the earthquake triggering^10,11,14^. These waves frequency is higher than the Brunt Vaisala frequency ~ 2.9 mHz^13^. The IGW_tsuna_ is the internal-gravity wave coupled with the tsunami propagation off-shore. These waves appear in the ionosphere after ~ 40 min of the rupturing. The frequency of these waves is lower than Brunt Vaisala frequency. These waves propagate with a speed of around 200–300 m/s^9,11–18^. The AW_Rayleigh_: is the acoustic wave coupled with the propagation of the seismic Rayleigh wave. These waves have two main frequencies of 3.68 mHz and 4.44 mHz and horizontal speed range from ~ 2.5 to 3.5 km/s^11,19,20^. Though, AW_Rayleigh_ is close to the acoustic wave speed, the corresponding time to reach the ionospheric layers is in the order of 10–15 min. These waves are essentially observed by GNSS-TEC and have been reported in several investigations in the past during the tsunami events^10,11,21,22^. These changes can be detected by GPS instrumentation^2,6,7,9,10,13,14,23^. The identification of these coupled seismo-ionospheric induced signals are essential from a scientific point of view and have been used to improve the tsunami early warning systems (TEWS)^24^. Manta et al.^13^ have proposed an empirical method to estimate displaced water volume during the tsunami-genesis by analyzing GPS derived AGW_epi_ and Rakoto et al.^12^ proposed a quantitative method to estimate the off-shore tsunami propagation amplitude by analyzing GPS derived IGW_tsuna_. In the near field, Hébert et al.^25^ proposed an integrated technique with buoy GPS, InSAR and pressure gauges anchored on the seafloor in the deep ocean to record tsunami initiation and propagation.

**Figure 1:**
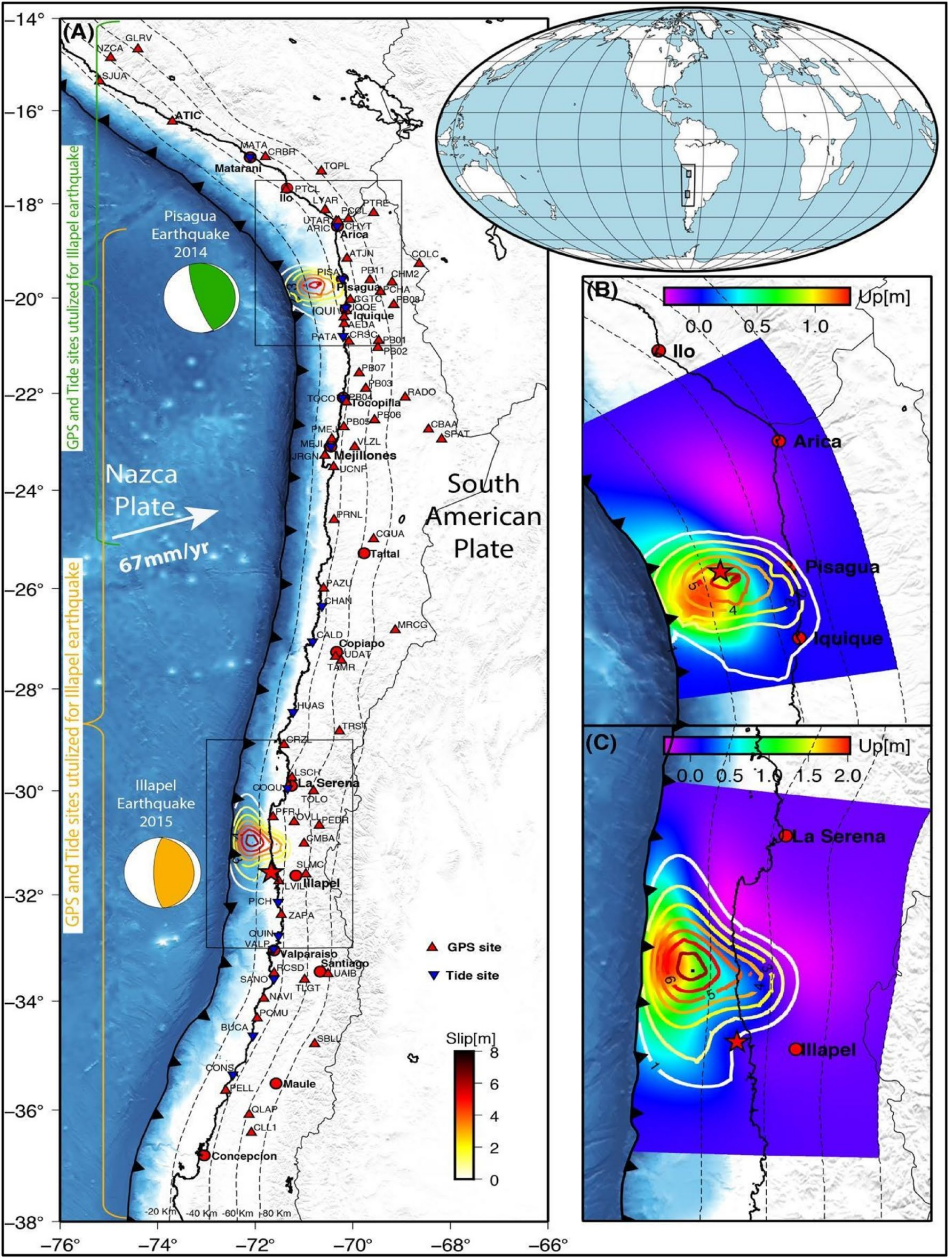
Chilean subduction zone and its tectonic setting. **(A)** The Chilean subduction zone experienced the 2014 Pisagua and the 2015 Illapel earthquakes. The focal mechanism solution of these earthquakes is shown in circles (green for the Pisagua earthquake and orange for the Illapel earthquake). The red triangles show the locations of GPS sites. The blue inverse triangles demonstrate the tide-gauge locations. The coseismic slip is demonstrated with the contour lines. The red stars indicate the earthquake epicenters. The rectangular region represents the area of the vertical deformation study. **(B,C)** The coseismic slip and vertical deformation produced by the Pisagua and Illapel earthquakes.

Herein, we correlated the variations of ionospheric TEC from the epicenter towards north and south of the GPS sites located around 1500 km along the Chilean coastal range and the tsunami propagation direction. We used two recent subduction earthquakes (Fig. 1) that occurred in northern Chile as case studies. Both earthquakes (the 2014 Pisagua earthquake M_w_ 8.1 (Fig. 1B) and the 2015 Illapel earthquake M_w_ 8.3 (Fig. 1C)) generated tsunamis, which are described in detail in the literature^26,27^. These two case studies were chosen as they possessed good-quality GPS data and tide-gauge records, allowing us to give a unique opportunity to investigate the components of surface deformation, tsunami propagation, and associated ionospheric TEC perturbations altogether in terms of AGW_epi_, AW_Rayleigh_ and IGW_tsuna_.

The Chilean subduction zone forms part of the Ring of Fire, hosting most of the largest earthquakes in the world. In particular, Chile’s west coast is characterized by the subduction of the Nazca Plate beneath the South American Plate, resulting in frequent and large megathrust earthquakes^28^. The Nazca Plate subducts east-northeast at a rate of approximately 67 mm/year^29^. Thus, this region yet again experienced two more tsunamigenic earthquakes: the 2014 Pisagua M_w_ 8.1 and the 2015 Illapel M_w_ 8.3^26,27,30–34^.

Several seismic and aseismic signals are transmitted during earthquakes (e.g., seismic waves, acoustic waves, gravity waves) towards the atmosphere^4^. The ionosphere forms part of the Earth’s atmosphere and is located at an altitude of approximately 100–1000 km. It contains ionized gas, called plasma, which influences radio wave propagation^35^. The ionosphere grows and shrinks depending on the energy it absorbs from the top sources (e.g., the sun, interplanetary medium, magnetosphere) and the bottom sources (e.g., mesosphere, stratosphere, troposphere, lithosphere). The lithospheric disturbances are predominantly caused by natural sources (e.g., earthquakes, volcanic eruptions, cryospheric changes)^36–40^ or human activity (e.g., nuclear explosions)^41^. The ionosphere is also highly influenced by large-scale tropospheric weather systems^35,42,43^, geomagnetic, auroral activity^43–45^, earthquakes^3–5,36^ and Solar eclipse^46^. During an earthquake, the ionosphere is mainly perturbed by AGW_epi_, acoustically induced AW_Rayleigh_, and tsunamis provoked IGW_tsuna_. Since, we observed three signals in the TEC disturbance field (i.e., two related to the earthquake rupture and the one linked to the tsunami propagation), along with the recent investigations^12,13,25^ our results from seismic, geodetic and oceanic tide gauge will significantly improve the existing TEWS^24^.

## Results

### Surface deformation

The 2014 Pisagua earthquake M_w_ 8.1 ruptured a segment of the Nazca‐South America subduction zone with the maximum coseismic slip concentrated in a single patch, and a total rupture extending for approximately 100 km along-strike and 130 km downdip^30,31,34,47^. According to the available slip models of the event, the peak coseismic slip of the mainshock occurred at 19.7°S–70.8°W, at a depth of approximately 23 km. The modeled vertical displacement of the Pisagua earthquake inferred from GPS data inversion is shown in Fig. 1B. The maximum uplift of approximately 1.20 m was in the southwestern part of the earthquake epicenter, and the maximum subsidence equaled approximately 0.25 m, being detected north of the epicenter. No vertical deformation occurred in the coastal region south of the main slip region, but minimum subsidence was detected north of the rupture. The 2015 Illapel earthquake M_w_ 8.3 also had the maximum coseismic slip concentrated in a single patch in the northern and western parts of the earthquake epicenter. The slip extended to the north and south of the main slip region. The interplate contact beneath the coastal region also slightly slipped^30,31,48^. Maximum uplift of approximately 2 m occurred near the trench, and maximum subsidence of 0.2 m was detected to the northeast of the maximum slip region (see Fig. 1C).

### Tsunami signature

**Figure 2:**
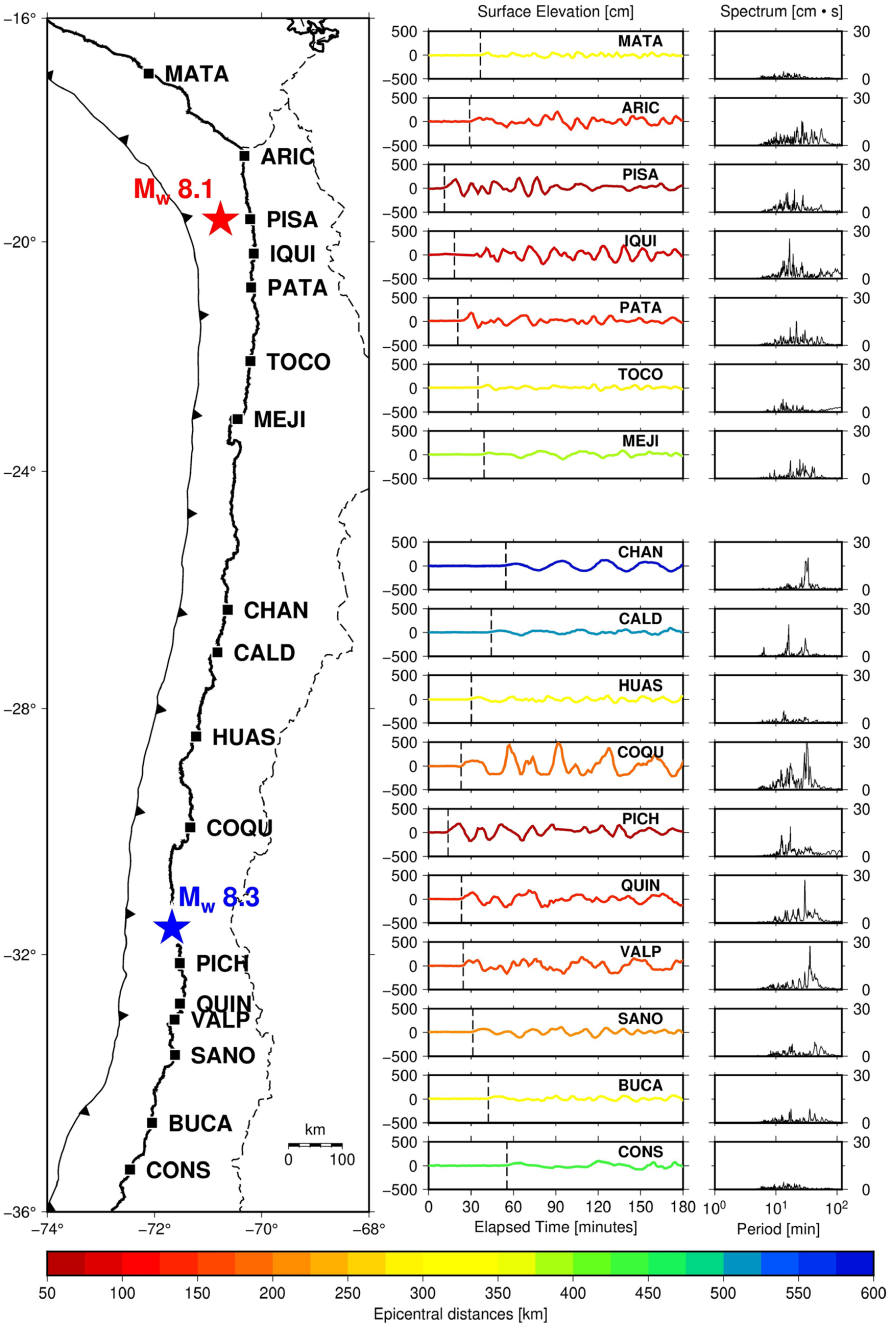
Tide-gauge locations along the Chilean coastal region (left panel). The tsunami waveform for the Pisagua and Illapel earthquakes are shown in central panel. The data were filtered using a zero-phase digital high-pass filter to eliminate signals over 180 min period. The dotted lines show the first arrival of tsunamis to the tide-gauge sites. Tsunami spectra of 24 h of elapsed time are shown in right panel.

The Pisagua earthquake M_w_ 8.1 produced a minor tsunami impact on the coastal area between Matarani (~ 17° S) and Mejillones (~ 23° S)^9^. The first wave arrived at Pisagua (~ 19.5° S) 11.08 min after the mainshock (Table S2). The area located between Arica (~ 18.5° S) and Patache (~ 20.8° S) presented the maximum range of wave height (approximately 1.5 m) (Fig. 2). North of Arica, the wave height decreased by almost 68% relative to the Arica tide gauge, whereas south of Patache, the wave height diminished by approximately 49% relative to the Patache tide gauge. On the contrary, the Illapel earthquake M_w_ 8.3 produced a substantial tsunami impact on the coastal area between Chañaral (~ 26° S) and Constitución (~ 35° S). The first wave arrived at Pichidangui (~ 32° S) 13.70 min after the mainshock (Table S2). The area located between Coquimbo (~ 30° S) and Valparaíso (~ 33° S) recorded the maximum range of wave height (over 1.5 m) (Fig. 2). The city of Coquimbo experienced massive flooding and flow height generated by a coastal harbor resonance process^25^, this fact is also demonstrated by the tsunami spectrum, which shows large energy at Coquimbo area (Fig. 2). North of Coquimbo, the wave height diminished by approximately 86% relative to the Coquimbo tide gauge, and south of Valparaíso, the wave height decreased by almost 42% relative to the Valparaíso tide gauge.

### TEC estimates

**Figure 3:**
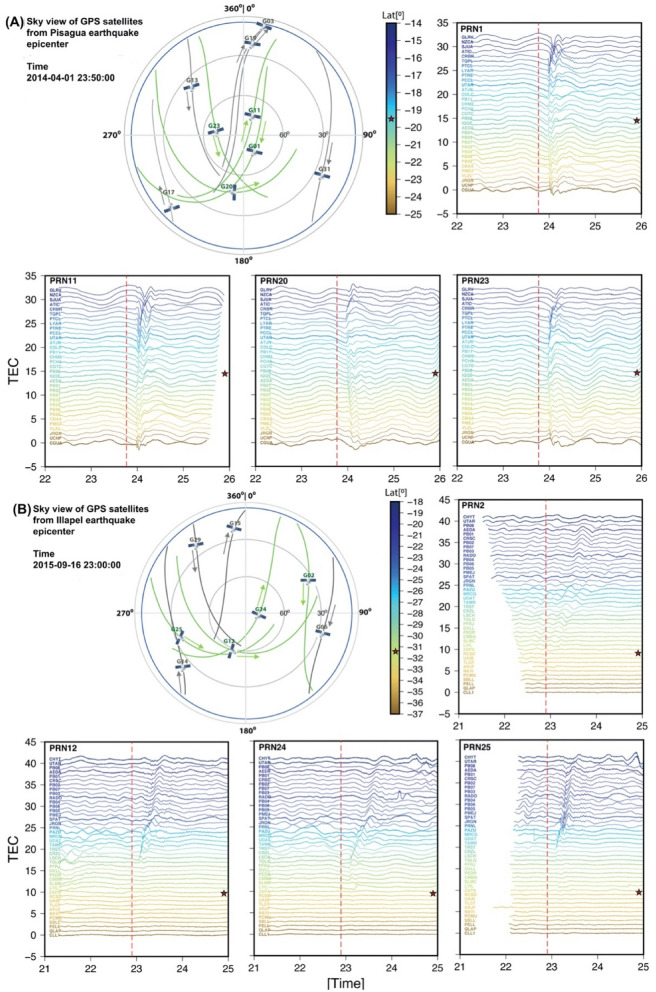
Imaginary sky view of the GPS satellites from the 2014 Pisagua earthquake **(A)** and Pisagua earthquake **(B)** epicenters. The blue circle represents the 20° elevation, which was used as criteria for the utilization of the GPS satellite data. The residual VTEC is presented for PRNs 01, 11, 20, and 23 during the 2014 Pisagua earthquake (**A**) and for PRNs 2, 12, 24, and 25 during the 2015 Illapel earthquake (**B**). The dotted lines mark the time of the earthquakes. The colored lines represent the residual VTEC regarding the locations of GPS latitudinally from north to south. The red stars indicate the latitude of the earthquake epicenters.

TEC over the epicentral region was influenced by surface uplift during both, the Pisagua and Illapel earthquakes (Figs. 1 and 3). During the Pisagua earthquake, 33 GPS sites located along the coastal region in northern Chile and southern Peru detected the ionospheric TEC variations (Fig. 1). The satellite Pseudo-Random Numbers (PRNs) 01, 11, 20, and 23 had sufficient coverage to identify changes in TEC. Three PRNs (13, 17, and 31) partially covered the Pisagua earthquake (Fig. 3A). The remaining TEC change data of the Pisagua earthquake are provided in Supplementary Information Fig. S1. PRNs 01, 11, 20, and 23 recorded significant TEC changes of approximately 1.25 TECU to the north and south of the rupture zone along the coast of northern Chile and southern Peru. Although PRNs 01, 11, and 23 were slightly above the 60° elevation, they could still detect the maximum changes of TEC (1.25 TECU) in the ionosphere (Fig. 3A). Some of the GPS-derived TEC results of Pisagua earthquake have been published by Reddy et al.^49^ and He and Heki^50^.

As for the Illapel earthquake, we were able to estimate TEC changes from 42 GPS sites. Upon observation that the TEC changes predominated in the northern direction, we selected the GPS sites to the north until Arica and to the south until Concepción. The observed TEC changes were significant (approximately 1.40 TECU) in the northern direction and negligible (approximately 0.35 TECU) in the southern direction. The TEC changes seemed to be unidirectional and not concentric, contrary to the case of the Pisagua earthquake. Astafyeva et al.^51^ suggested that properties (e.g., amplitude, waveform etc.) of ionospheric disturbances caused by seismic activity are dependent on the observational factors, the current geometry of GNSS-sounding, background ionization and current geomagnetic field. Further, they discussed the effect of earth’s magnetic field configuration at the epicenter area, which controls propagation of the co-seismic ionospheric disturbances in the specific direction based on the results of Rolland et al.^10^. Rolland et al.^10^ observed minimum change in the co-seismic ionospheric disturbances above and south of the rupture line in the northern hemisphere, whereas minimum changes are observed north of the rupture line in the southern hemisphere. They have discussed the example of M_w_ = 8.8 2010 offshore Maule Chile earthquake, for which they observed negative initial change in GPS-TEC observations south of rupture line and positive initial change north of rupture line. The presence of unidirectionality in the TEC observations in the case of the Illapel earthquake seems to follow the effect of the geomagnetic field. Furthermore, Reddy et al.^52^ and Ravanelli et al.^14^ also observed similar unidirectionality in the TEC for Illapel earthquake. The TEC changes detected by PRNs 12 and 25 at elevations ranging from 30° to 60° were characterized by a wavy signal. Other PRNs (2 and 24) did not exhibit any wavy signal (Fig. 3B). The remaining TEC change data of the Illapel earthquake are provided in Supplementary Information Fig. S2.

Changes in TEC were clearly observed during the Pisagua and Illapel earthquakes at different amplitudes. The changes were systemically symmetric at the GPS sites to the north and south of the Pisagua rupture zone. Around 700 s after the earthquake initiation, the TEC changes started to diminish, and within the next 100 s, we detected an increase in the TEC changes. A similar scenario was noted both in the northern and southern directions. Conversely, during the Illapel earthquake, approximately 600 s after the earthquake initiation, we observed a decline and a subsequent increase in the TEC perturbations. The TEC perturbations were observed only to the north of the Illapel rupture and were not recorded in the southern direction (Fig. 3B).

## Discussion

The determination of the intensity and amplitude of tsunami and its propagation depends on the initial sea-surface deformation induced by the seabed due to the occurrence of slip along the plate interface, the accompanying water column excitation^53^ and bathymetry^54^. The simulation of tsunami propagation in real-time has been attempted by several researchers via automated finite-fault inversion^55,56^ and fast numerical propagation and inundation models, such as HySEA^57^. Despite these scientific efforts, there is still a high level of uncertainty and low level of accuracy in the estimation of tsunami hazards^58^. Nowadays, the acquisition of the sea level variation in real-time at the earthquake source is only available for submarine sensors networks^59,60^ (e.g., DONET in Japan and NEPTUNE in Canada). In the analyzed area, Chile has an extensive real-time tide gauges network that provides quality information of tsunami coastal propagation. Nevertheless, the tsunami dynamics have to be detected accurately, remotely and redundantly whenever direct observations are not feasible in order to develop a reliable real-time tsunami warning system.

Herein, with the previous works^12,13^ and the present analysis, we discuss that space-based geodetic GPS signals can be used to continuously monitor and sense the tsunami signals along with the possibility to estimate amplitude and the volume of displaced water during Tsunami. Thus, enhancing the possibility of tsunami detection in such tectonically active regions as the Chilean subduction zone.

### Surface deformation and tsunami propagation

The finite-fault model of the Pisagua and Illapel earthquakes provided details on the vertical deformation caused by both earthquakes (Fig. 1). In the Pisagua region, the maximum vertical uplift was circular and near the epicenter. However, in the Illapel region, the vertical uplift was elliptical and expanded to the north and south from the main uplift peak (Fig. 1). The characteristics of the epicenter and main peak of coseismic slip suggested that, during the Pisagua earthquake, the rupture propagated up-dip towards the west and slightly south. On the contrary, during the Illapel earthquake, the deeply locked zone propagated up-dip and northwest, toward the trench (Fig. 1). The tsunami wave height during the Pisagua earthquake was less than that during the Illapel earthquake (Fig. 2). The Pisagua earthquake-associated tsunami extended to the north and south with almost the same wave height. However, the tsunami associated with the Illapel earthquake propagated only to the north. The finite-fault model^16^ suggested that the rupture was initiated near the coast of Illapel and propagated northwestward, presenting a possible reason for the northward tsunami propagation. This explanation is supported by the observed tide-gauge data from the Coquimbo coastal region (Fig. 2). Thus, crustal uplift and tsunami propagation act as bottom sources of ionospheric TEC perturbations.

### Relationship between TEC and tsunami propagation

**Figure 4:**
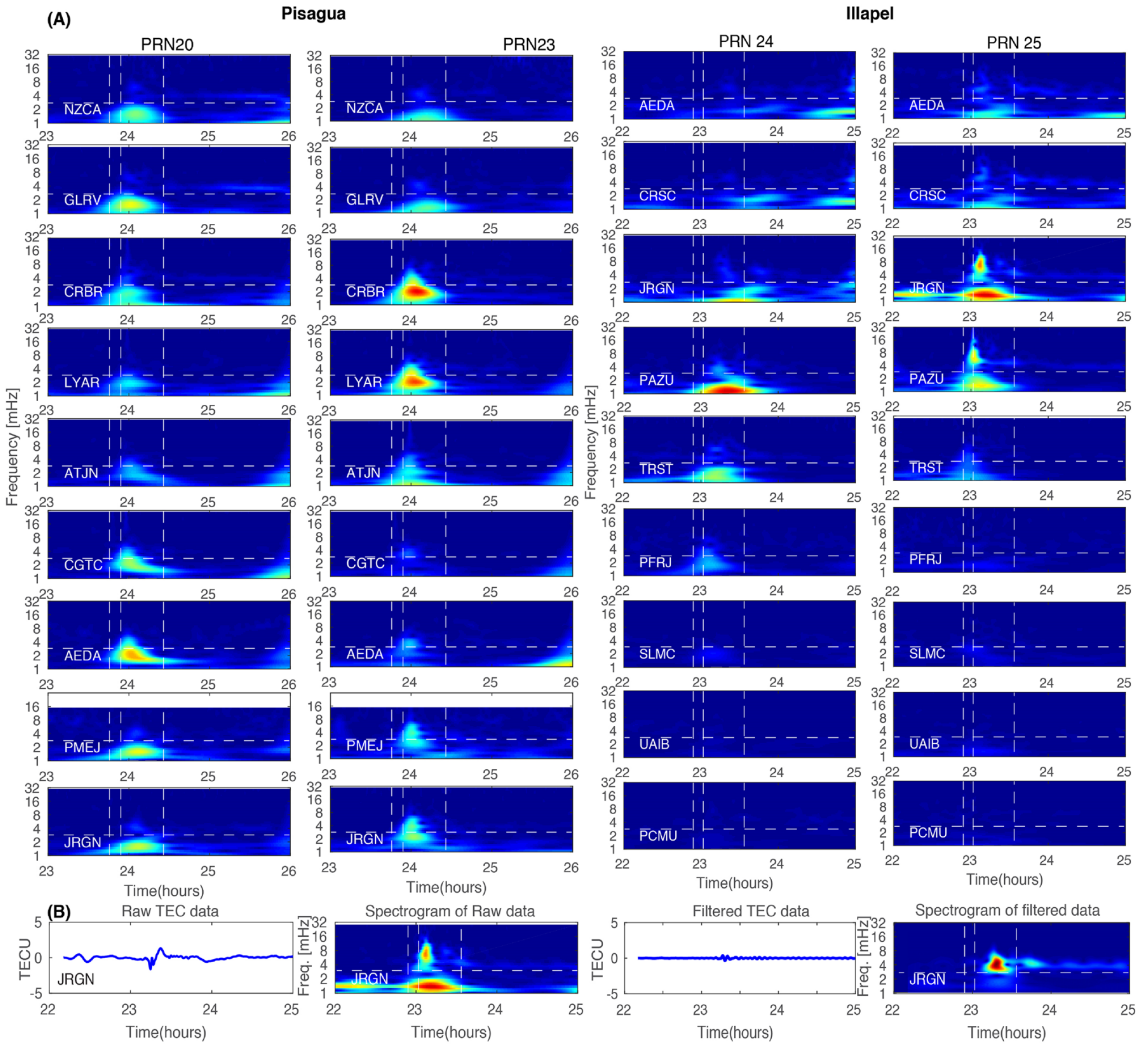
**(A)** Spectrogram of the residual TEC changes at randomly chosen GPS sites for PRNs 20 and 23 for the Pisagua and PRNs 24 and 25 for the Illapel earthquakes. The first dotted line represents the timing of the earthquakes; the second dotted line demarcates 8 min after the initiation of the earthquakes; and the third dotted line represents 40 min after the initiation of the earthquakes. **(B)** To identify the AW_Rayleigh_**,** the raw and filtered TEC of JRGN GPS site of PRN25 along with spectrogram provided.

**Figure 5:**
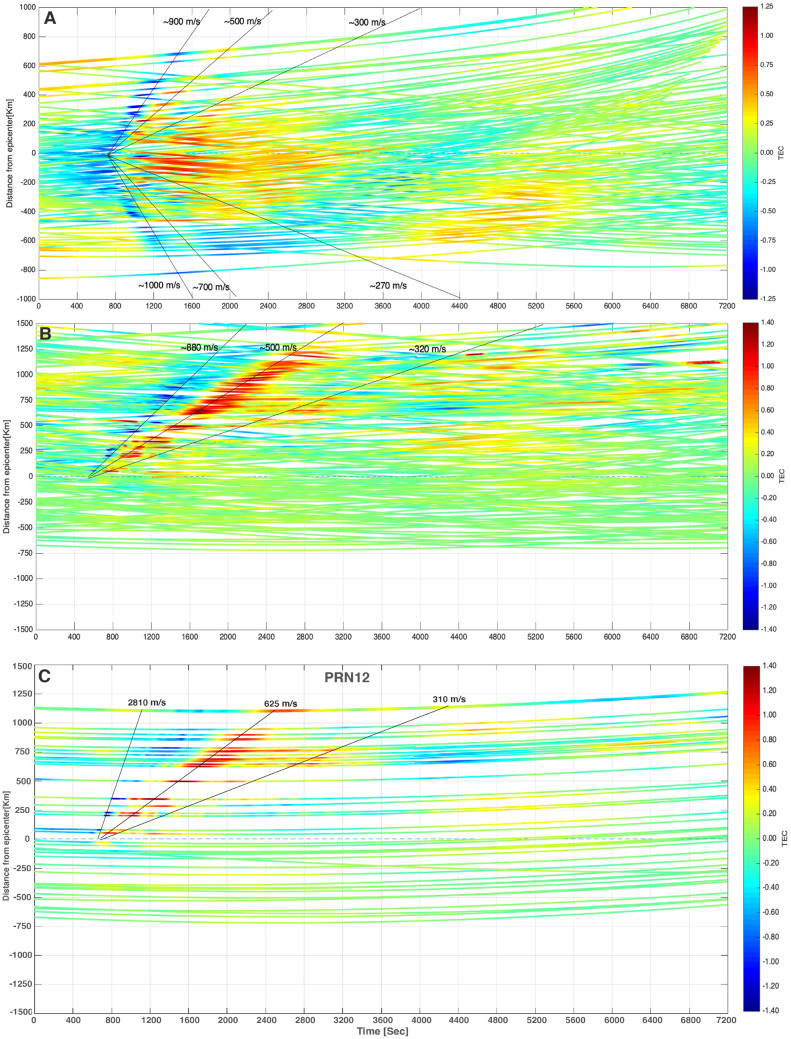
**(A)** Hodochrone map shows variations in VTEC for PRNs 01, 11, 20, and 23 for GPS sites for two hours duration from 23 to 1 h next day of the Pisagua earthquake on April 1, 2014. **(B)** Hodochrone plot shows residual VTEC at various GPS sites as a function of time and epicentral distance, as obtained from PRNs 2, 12, 24, and 25 for GPS sites for two hours duration from 22 to 24 h of the Illapel earthquake on September 16, 2016. The positive and negative epicentral distances indicate the northern and southern directions regarding the Illapel earthquake epicenter, respectively. **(C)** The signature of AW_Rayleigh_ is shown in TEC data of PRN12 in the hodochrone.

To better understand the effect of two seismic events (i.e., the Pisagua and Illapel earthquakes) on the TEC signature, we performed the wavelet analysis of TEC data. For the analysis, we selected two pairs of GPS, i.e., PRNs 20 and 23 for the Pisagua earthquake and PRNs 24 and 25 for the Illapel earthquake, for all GPS sites. During the Pisagua event, the TEC values presented a perturbation attributed to AGW_epi_ and IGW_tsuna_. These waves had a frequency of 1–7 mHz at around 700 s after the mainshock and propagated away from the epicenter with a horizontal speed of 500–700 m/s. Subsequently, a low-frequency wave related to IGW_tsuna_ was originated from the tsunami and traveled at a speed of 300 m/s. As for the Illapel earthquake, the TEC signature remained intense and dominant in the northern direction. This signature has also been observed by Reddy et al.^51^ and Ravanelli et al.^14^. The spectral analysis of the TEC (Fig. 4A) observed during the Pisagua and Illapel earthquakes revealed the unique characteristics of their AGW_epi_ and IGW_tsuna_. The TEC traces depicted in the hodochrone of Fig. 5A,B indicate that the satellite coverage was dense enough to assure the detection of AGW_epi_ and IGW_tsuna_. For identifying the AW_Rayleigh_, we have used band pass filter 3–7 mHz in the TEC data of PRN25. The raw and filtered TEC data are provided in Fig. 4B along with spectrograms. It clearly represents the AW_Rayleigh_ and AGW_epi_ signatures. Further to show the TEC perturbation akin to the AW_Raylaigh_, we provided a hodochrone of PRN12 in Fig. 5C. which clearly portrays the AW_Raylaigh_ allied TEC perturbation before AGW_epi_ with a velocity of ~ 2810 m/s in the far field. The remaining TEC traces associated to AW_Rayleigh,_ AGW_epi_, and IGW_tsuna_ are presented in the hodochrone of Supplementary Information Figs. S3 and S4.

As previously proposed, three major mechanisms can explain ionospheric perturbations during earthquakes and associated tsunamis in the form of TEC changes by producing (i) AGW_epi_waves^6,10,11,14,61,62^, (ii) AW_Rayleigh_ by surface Rayleigh waves^10,11,19,20^, and (3) IGW_tsuna_ by tsunami waves^9,11–18,63–65^. The propagation of seismo-acoustic waves in the atmosphere and their interactions with the ionospheric plasma are crucial for TEC variations^3–5^. Herein, the TEC perturbation patterns for both earthquakes significantly differed owing to the AGW_epi_ wave propagation directions. The TEC changes were nearly uniform for all satellites during the Pisagua earthquake (Fig. 3) and substantially varied with changing elevation of moving GPS satellites during the Illapel earthquake (Fig. 3).

The AGW_epi_ waves are formed not only due to the uplift of the ocean/land surface but also due to the kinematic uplift of the water column. The water column and crustal uplift depend on the rupture propagation direction and slip appearance during earthquake generation. The TEC changes derived from the GPS signals enable the identification of the rupture propagation direction. The direction of tsunami propagation is affected not only by static slip but also by kinematic slip generation^66^. The crust surface uplift provides direct uplift of the water column and subsequent tsunami propagation^67^. The earthquake focal mechanism, the associated direction of rupture propagation, and the earthquake magnitude are equally important in the manifestation of ionospheric responses^21^. Therefore, the TEC changes identified via ground-based GPS sites in the coastal region can be utilized to detect tsunamis in subduction zones in real-time.

### Potential of AW_Rayleigh_ waves for tsunami detection

GPS satellites PRNs 02 and 24 passed through elevation angles of approximately 30° and over 70°, respectively (Fig. 3A,B). Both of these satellites detected the same range of perturbations in the TEC signals. However, PRNs 12 and 25 passed between the 30° and 60° elevation angles and detected wavy signals in the TEC perturbation. Rolland et al.^38^ suggested that satellites with elevation angles lower than 40° could only provide the efficient detection of AW_Rayleigh_ wave-induced ionospheric waves. However, as shown in Fig. 3, the TEC of PRNs 12 and 25 shows a minor wavy signal that appears before the main TEC perturbation and continues after the inclination in the far field. This wavy signal can be potentially utilized for tsunami detection in tsunami warning systems in the future.

**Figure 6:**
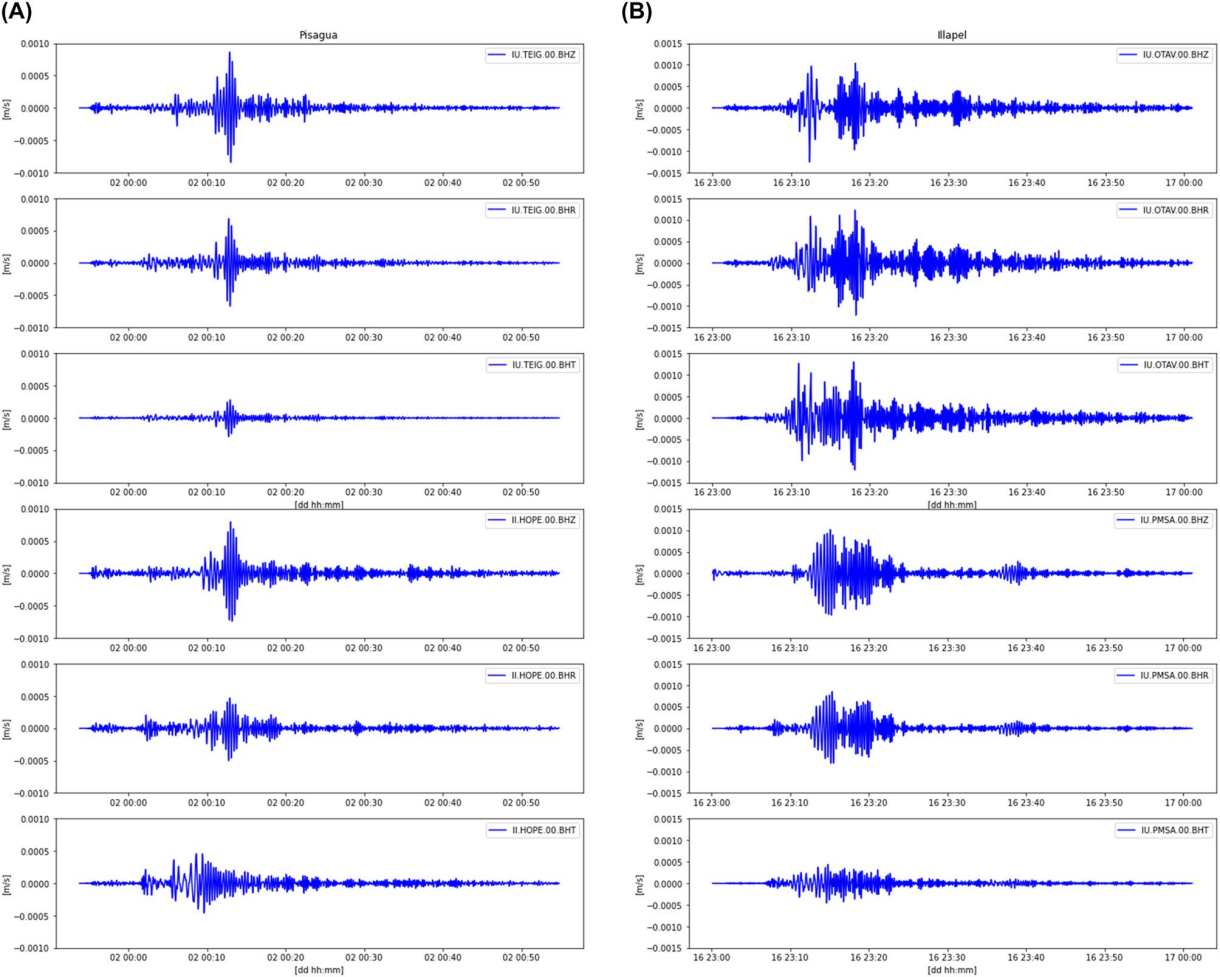
**(A)** Propagation of Raleigh wave at seismic sites TEIG (to the north) and HOPE (to the south) of the epicenter. These seismic sites are situated approximately 43° away from the Pisagua earthquake epicenter. **(B)** Propagation of Raleigh wave at seismic sites OTAV (to the north) and PMSA (to the south) of the epicenter. These seismic sites are located approximately 33° away from the Illapel earthquake epicenter.

To verify the presence of AW_Rayleigh_ waves in the TEC perturbation during the Pisagua and Illapel earthquakes, we checked the vertical component of seismic Rayleigh waves in the seismic sites situated to the north and south of both rupture zones. The positions of chosen seismic sites are presented in Table S3. Figure 6A,B demonstrates that Rayleigh waves were present in both the Pisagua and Illapel earthquakes. During the Pisagua earthquake, the dispersion of Rayleigh waves was produced in the northern and southern directions, and they had similar energy. The values for the amplitude of the Z and R components from station TEIG (located to the north of the hypocenter) were similar to those from station HOPE (located to the south of the hypocenter) (Fig. 6 and Table S3). A dissimilar pattern in the dispersion of Rayleigh waves was observed during the Illapel earthquake, with different amplitudes of the Z and R components. More intense Rayleigh wave dispersion was detected at the OTAV station (located to the north of the Illapel hypocenter) rather than at the PMSA station (located to the south of the hypocenter). These observations coincide with the TEC perturbations estimated from the GPS data and indicate that Rayleigh wave propagation during the Illapel earthquake was directed to the north and dispersion to the south. This also explains why Rayleigh wave dispersion to the south was not detected. As a result, the identification of ionospheric perturbations from coastal GPS sites may be a reliable and cost-effective technique to be applied to TEWS. Hence, the GPS-derived TEC methodology can be utilized as it avoids the costs of the new monitoring equipment installation and implementation by using pre-installed GPS sites.

## Concluding remarks

Seismic instruments (e.g., ocean buoys, pressure sensors) installed on the ocean bottom and real-time tsunami finite-fault rupture models are currently used to forecast the possible propagation direction of tsunami waves. The research presented herein demonstrates that GPS-derived TEC could also be effectively utilized to improve the existing TEWS. In Chile’s coastal region, early tsunami detection is possible via the rapid determination (5–10 min) of changes in TEC using the available GPS sites. Earthquake ruptures significantly displace the ocean surface and create waves (AGW_epi_, IGW_tsuna_ and AW_Rayleigh_), which reach the ionosphere and cause measurable changes in ionospheric electron density. The joint analysis of seismic events and changes in TEC can substantially improve tsunami detection not only in the Chile subduction zone but also in other tsunami-prone areas worldwide.

## Methods and data

Tsunami signals were obtained using remotely accessed GPS data from most Chilean and Peruvian coastal GPS sites. These GPS sites were operated continuously with a sampling rate of 15 s. The propagation direction of the tsunami waves was detected by the tide-gauge sites situated to the north and south of the rupture zone.

### Vertical deformation associated with the Pisagua and Illapel earthquakes

The vertical deformation of a megathrust earthquake depends on the coseismic slip pattern and locations in the plate interface. Most of the subduction earthquakes, especially in Chile, occur in the seismo-genic zone 20–60 km deep^69^. The vertical deformations produced during the Pisagua and Illapel earthquakes were modeled following the dislocation theory by Okada^70^. We also used the finite-fault model for the 2014 Pisagua and the 2015 Illapel earthquakes, as suggested by Shrivastava et al.^31,32^. The modeled vertical displacements for the Pisagua and Illapel earthquakes are shown in Fig. 1B,C.

### TEC derived from GPS data

The ionosphere serves as a dispersive medium for the propagating GPS signals and reduces the GPS signal velocity. The dispersive potential of TEC can be estimated using the L1 and L2 frequencies of GPS signals. The GPS TEC analysis provides the vertical total electron content (VTEC) estimated by the techniques suggested by Rama Rao et al.^71^ and Seemala and Valladares^72^. VTEC is defined as the line integral of the electron density from all GPS satellites visible from each of the receivers above a user-specified elevation cut-off angle (we used 20°). In the GPS system, every satellite transmits signals at two frequencies: *f*_1_ = 1575.42 MHz and *f*_2_ = 1227.60 MHz.

1 $$STEC = \frac{{2(f_{1} f_{2} )^{2} }}{{k\left( {f_{1}^{2} - f_{2}^{2} } \right)}}\left( {P_{2} - P_{1} } \right) + \tau ^{r} + \tau ^{s} ,$$

where *k* is the ionosphere refraction and equals 80.62 (m^3^/s^2^); *P*_*1*_ and *P*_*2*_ are the pseudoranges; and *τ*^*r*^ and *τ*^*s*^ are the differential code biases corresponding to pseudoranges *P*_1_ and *P*_2_.

VTEC (in el/m^2^) can be computed after Ma and Maruyama^73^ as follows:

2 $$VTEC = STEC~ \times ~\cos ~\chi ,$$

where $\chi$ is the zenith angle and can be expressed as:

3 $$\chi = \arcsin \left( {\frac{{R_{E} \text{Cos} ~\alpha }}{{R_{E} + h}}} \right).$$

We analyzed VTEC at different sets of GPS stations along the coastal region for two recent Chilean subduction earthquakes: the 2014 Pisagua and the 2015 Illapel. The Pisagua earthquake took place on April 1, 2014, at 23:47 UT in northern Chile, and the Illapel earthquake occurred on September 16, 2015, at 22:54 UT in central Chile. For TEC analysis, we used the GPS site data (Table S1) and considered the satellite data up to the 20° elevation. During the Pisagua earthquake (from its epicenter to the 20° elevation in the sky), seven satellites were available, including PRNs 01, 11, 13, 19, 20, 23, and 31. Within the first 10 min after the earthquake initiation, PRN 19 went below the 20° elevation, and no other satellite vehicle appeared in the 20° elevation region. However, satellite PRN 17 appeared 20 min later. Therefore, we utilized seven PRNs (i.e., 01, 11, 13, 17, 20, 23, and 31) for VTEC analysis. To detect changes in VTEC associated with the Pisagua earthquake, the VTEC data were analyzed 2 h before and 2 h after the earthquake. As the Pisagua earthquake occurred at 23:47 UT, in order to obtain complete coverage of 2 h after the earthquake, we added the TEC data for April 1–2, 2014. Thus, we chose PRNs 01, 11, 20, and 23, which provided good coverage from 22:00 UT to 02:00 UT for April 1–2, 2014.

A similar analysis was performed for the Illapel earthquake. Our choice of GPS stations is shown in Table S1. During the Illapel earthquake (from its epicenter to the 20° elevation in the sky), six satellites were available, including PRNs 02, 06, 12, 15, 24, and 25. Within the first 10 min after the earthquake initiation, PRN 15 went below the 20° elevation, whereas PRNs 14 and 29 went above the 20° elevation region. Therefore, seven PRNs (i.e., 02, 06, 12, 14, 24, 25, and 29) were utilized for TEC analysis. PRNs 02, 12, 24, and 25 were chosen as they fully covered the earthquake timing (at 22:54 UT) from 21:00 UT to 24:00 UT.

To better visualize the effect of the earthquake and tsunami on the TEC data, we estimated differential VTEC (DVTEC) by fitting a 7th order polynomial to the VTEC data for each station, following Ozeki and Heki^74^ and Lay et al.^75^. The 7th order polynomial was chosen because it provides the best fit with the VTEC data. This method proved to be effective and revealed the earthquake and tsunami effects, allowing us to remove only the diurnal periodic variations and leave the remaining signals with TEC changes. The final TEC changes associated with the Pisagua and Illapel earthquakes are illustrated in Fig. 3A,B, respectively.

### Sea-level record of tsunamis associated with the Pisagua and Illapel earthquakes

The sea-level records of the 2014 Pisagua and the 2015 Illapel earthquakes were extracted from the coastal tide-gauge network [http://www.ioc-sealevelmonitoring.org]. The tsunami records were processed through a zero-phase digital high-pass filter to eliminate signals over a 3-h period to filter out tidal variations^76^. This value is selected based on the results of Bai et al.^77^, thus only shelf and bay modes may be identified. As a matter of fact, previous results from Catalán et al.^24^ showed that most important resonant modes are lower than 120 min in northern Chile. Moreover, the tsunami spectra were computed from 24 h of elapsed time once arrival.

### Detection of AW_Rayleigh_ waves using Pisagua and Illapel seismic waveforms

The seismic waveforms and instrumental responses were obtained from the IRIS website (IRISDMC, http://ds.iris.edu/wilber3/find_event). The metadata for the selected stations is provided in Supplementary Information Table S3. To detect the Pisagua and Illapel AW_Rayleigh_ from the seismic waveform data, we considered a spectral bandwidth of 0.02–0.2 Hz. We applied the 10th order Butterworth bandpass filter to remove the instrumental response between the frequencies mentioned above. The corrected and filtered waveforms were rotated to the ZNE coordinate system (Z, North, East). To analyze the AW_Rayleigh_ amplitudes with more precision, a new rotation was performed from the NE (northeast) to RT (radial transverse) directions. The AW_Rayleigh_ were principally analyzed in the Z (vertical) and R (radial) directions, and the amplitudes were in velocity units (m/s).

## Supplementary Information

Supplementary Information.
