# Supplementary material for: Tsunami detection by GPS-derived ionospheric total electron content
Source: Sci Rep. 2021 Jun 21;11:12978. doi: 10.1038/s41598-021-92479-3 (PMC8217264; doi:10.1038/s41598-021-92479-3)
Supplement: Supplementary file 1 — Supplementary Information. [file 41598_2021_92479_MOESM1_ESM.docx]

**Supplementary Information**

**Tsunami detection by GPS-derived ionospheric total electron content**

### Mahesh N Shrivastava^1,2^*, Ajeet K Maurya^3^, Gabriel Gonzalez^1,2^, Poikayil S Sunil^4^, Juan Gonzalez^2,5^, Pablo Salazar^1,2^ and Rafael Aranguiz^2,5^

^1^Department of Geological Sciences, Universidad Catolica del Norte, Antofagasta, Chile

^2^National Research Center for Integrated Natural Disaster Management, Santiago, Chile

^3^Department of Physics, Doon University, Dehradun, India

^4^Department of Marine Geology and Geophysics, School of Marine Sciences, Cochin University of Science and Technology, Kochi, India

^5^Department of Civil Engineering, Universidad Catolica de la Santísima Concepcion, Chile

*E-mail: [mahesh.shrivastava@ucn.cl](mailto:mahesh.shrivastava@ucn.cl)

**Contents of this file**

Table S1 to S3

Figure S1 to S4

**Supplementary Table S1**. GPS sites in Pisagua and Illapel Earthquake region

| **Pisagua**  **Earthquake** | | | | **Illapel**  **Earthquake** | | | |
| --- | --- | --- | --- | --- | --- | --- | --- |
| **S/N** | **GPS sites** | **Latitude**  **(Degree)** | **Longitude**  **(Degree)** | **S/N** | **GPS**  **sites** | **Latitude**  **(Degree)** | **Longitude**  **(Degree)** |
| 1 | GLRV | -14.670 | -74.404 | 1 | CHYT | -18.370 | -70.342 |
| 2 | NZCA | -14.856 | -74.964 | 2 | UTAR | -18.375 | -70.296 |
| 3 | SJUA | -15.363 | -75.188 | 3 | PB08 | -20.143 | -69.161 |
| 4 | ATIC | -16.231 | -73.697 | 4 | AEDA | -20.546 | -70.177 |
| 5 | CRBR | -16.996 | -71.790 | 5 | PB01 | -20.898 | -69.470 |
| 6 | TQPL | -17.304 | -70.643 | 6 | CRSC | -20.917 | -70.079 |
| 7 | PTCL | -17.701 | -71.370 | 7 | PB02 | -21.044 | -69.487 |
| 8 | LYAR | -18.134 | -70.569 | 8 | PB07 | -21.577 | -69.869 |
| 9 | PTRE | -18.194 | -69.574 | 9 | PB03 | -21.898 | -69.734 |
| 10 | PCCL | -18.325 | -70.089 | 10 | RADO | -22.082 | -68.926 |
| 11 | UTAR | -18.375 | -70.296 | 11 | PB04 | -22.183 | -70.132 |
| 12 | ATJN | -19.164 | -70.119 | 12 | PB06 | -22.552 | -69.555 |
| 13 | COLC | -19.276 | -68.639 | 13 | PB05 | -22.698 | -70.185 |
| 14 | PB11 | -19.622 | -69.648 | 14 | PMEJ | -22.945 | -70.431 |
| 15 | CHM2 | -19.669 | -69.194 | 15 | SPAT | -22.952 | -68.178 |
| 16 | PCHA | -19.869 | -69.432 | 16 | JRGN | -23.288 | -70.574 |
| 17 | CGTC | -20.035 | -70.052 | 17 | PRNL | -24.604 | -70.384 |
| 18 | PB08 | -20.143 | -69.161 | 18 | PAZU | -25.996 | -70.599 |
| 19 | IQQE | -20.273 | -70.131 | 19 | MRCG | -26.835 | -69.130 |
| 20 | AEDA | -20.403 | -70.178 | 20 | UDAT | -27.357 | -70.354 |
| 21 | PB01 | -20.898 | -69.470 | 21 | TAMR | -27.436 | -70.235 |
| 22 | PB02 | -21.044 | -69.487 | 22 | TRST | -28.836 | -70.273 |
| 23 | PB07 | -21.577 | -69.869 | 23 | CRZL | -29.101 | -71.409 |
| 24 | PB03 | -21.898 | -69.734 | 24 | LSCH | -29.742 | -71.246 |
| 25 | PB04 | -22.183 | -70.132 | 25 | TOLO | -30.003 | -70.806 |
| 26 | PB06 | -22.552 | -69.555 | 26 | PFRJ | -30.506 | -71.635 |
| 27 | PB05 | -22.698 | -70.185 | 27 | OVLL | -30.603 | -71.203 |
| 28 | CBAA | -22.746 | -68.448 | 28 | PEDR | -30.670 | -70.689 |
| 29 | PMEJ | -22.945 | -70.431 | 29 | CMBA | -31.018 | -70.999 |
| 30 | VLZL | -23.110 | -69.960 | 30 | SLMC | -31.605 | -70.963 |
| 31 | JRGN | -23.280 | -70.570 | 31 | LVIL | -31.737 | -71.514 |
| 32 | UCNF | -23.520 | -70.392 | 32 | ZAPA | -32.378 | -71.466 |
| 33 | CGUA | -24.997 | -69.573 | 33 | RCSD | -33.477 | -71.613 |
|  | | | | 34 | UAIB | -33.493 | -70.509 |
|  |  |  |  | 35 | TLGT | -33.599 | -70.989 |
|  |  |  |  | 36 | ARJF | -33.628 | -78.836 |
|  |  |  |  | 37 | NAVI | -33.952 | -71.824 |
|  |  |  |  | 38 | PCMU | -34.317 | -71.961 |
|  |  |  |  | 39 | SBLL | -34.792 | -70.780 |
|  |  |  |  | 40 | PELL | -35.645 | -72.606 |
|  |  |  |  | 41 | QLAP | -36.084 | -72.125 |
|  |  |  |  | 42 | CLL1 | -36.411 | -72.080 |

**Supplementary Table S2.** Summary of arrival times and maximum wave height of tsunami associated to the Pisagua M_w_ 8.1 and Illapel M_w_ 8.3 earthquake.

| **Pisagua**  **Earthquake** | | | | | **Illapel**  **Earthquake** | | | | |
| --- | --- | --- | --- | --- | --- | --- | --- | --- | --- |
| **S/N** | **Tide gauge station** | **Arrival time [min]** | **Max. wave height [m]** | **Max. wave time [min]** | **S/N** | **Tide gauge station** | **Arrival time [min]** | **Max.**  **wave height [m]** | **Max. wave time [min]** |
| 1 | MATA | 36.59 | 0.62 | 104.80 | 1 | CHAN | 54.64 | 1.06 | 126.80 |
| 2 | ARIC | 29.07 | 1.96 | 89.20 | 2 | CALD | 44.34 | 1.05 | 524.61 |
| 3 | PISA | 11.08 | 2.19 | 79.03 | 3 | HUAS | 30.19 | 0.65 | 126.86 |
| 4 | IQUI | 18.19 | 1.83 | 138.31 | 4 | COQU | 22.99 | 4.69 | 90.87 |
| 5 | PATA | 20.64 | 1.69 | 30.24 | 5 | PICH | 13.70 | 1.75 | 21.77 |
| 6 | TOCO | 34.86 | 0.89 | 261.38 | 6 | QUIN | 23.28 | 1.74 | 71.08 |
| 7 | MEJI | 39.20 | 0.86 | 449.63 | 7 | VALP | 24.49 | 1.68 | 107.83 |
|  | | | | | 8 | SANO | 31.19 | 0.98 | 93.86 |
|  |  |  |  |  | 9 | BUCA | 42.22 | 0.72 | 335.12 |
|  |  |  |  |  | 10 | CONS | 55.31 | 1.24 | 191.29 |

**Supplementary Table S3.** Summary of arrival times and maximum wave height of tsunami associated to the Pisagua M_w_ 8.1 and Illapel M_w_ 8.3 earthquake.

|  | **Station Network** | **Lat. [^o^]** | **Long. [^o^]** | **Distance [°]** | **Azimuth [°]** | **Elevation (MSL)** | **Location** |
| --- | --- | --- | --- | --- | --- | --- | --- |
| PI SA  GUA | TEIG IU | 20.23 | -88.28 | 43.37 | -24.24 | 40 | Yucatan, Mexico |
|  | HOPE II | -54.28 | -36.49 | 43.38 | 151.38 | 20 | South Georgia Island |
| Ill  AP  EL | OTAV IU | 0.24 | -78.45 | 32.45 | -12.70 | 3510 | Ecuador |
|  | PMSA IU | -64.77 | -64.05 | 33.54 | 174.12 | 40 | Antarctic |


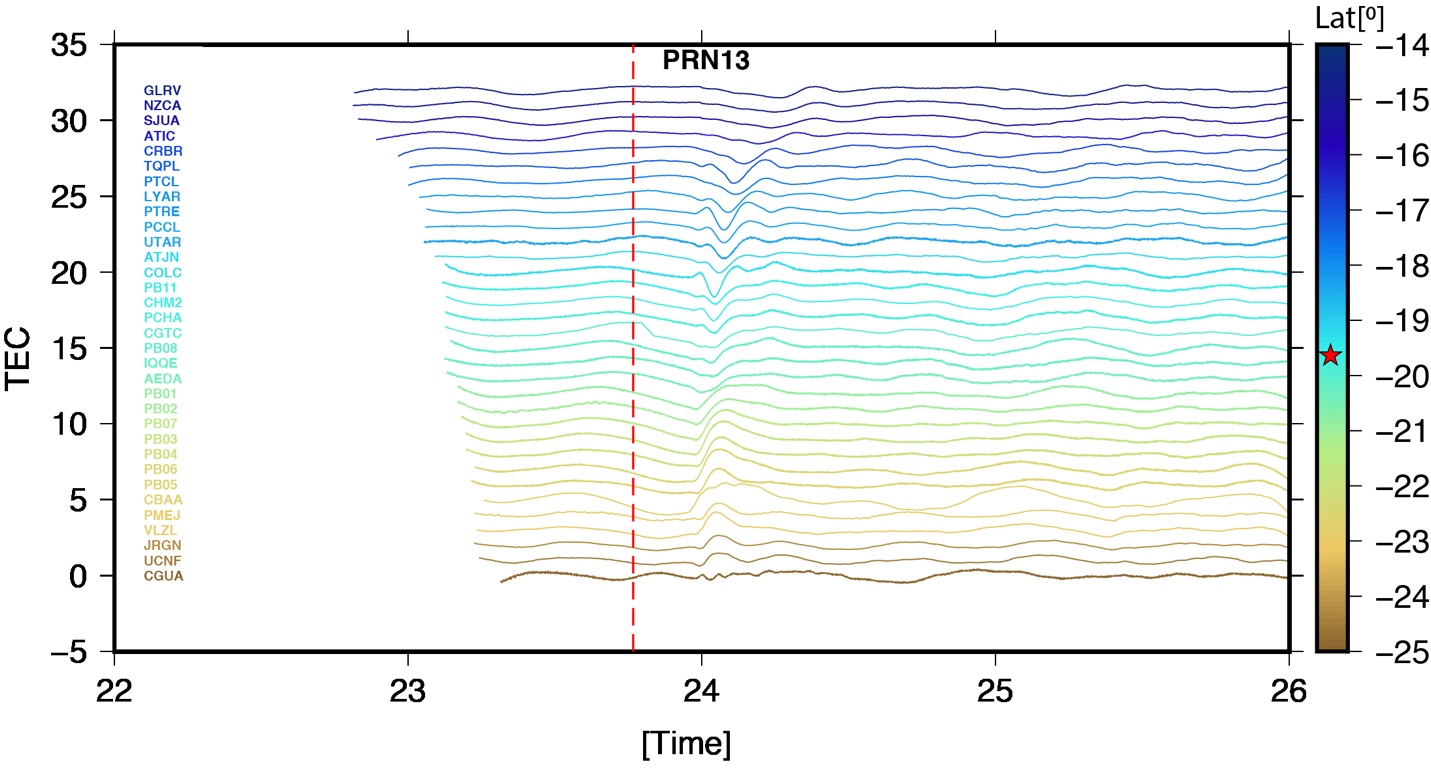


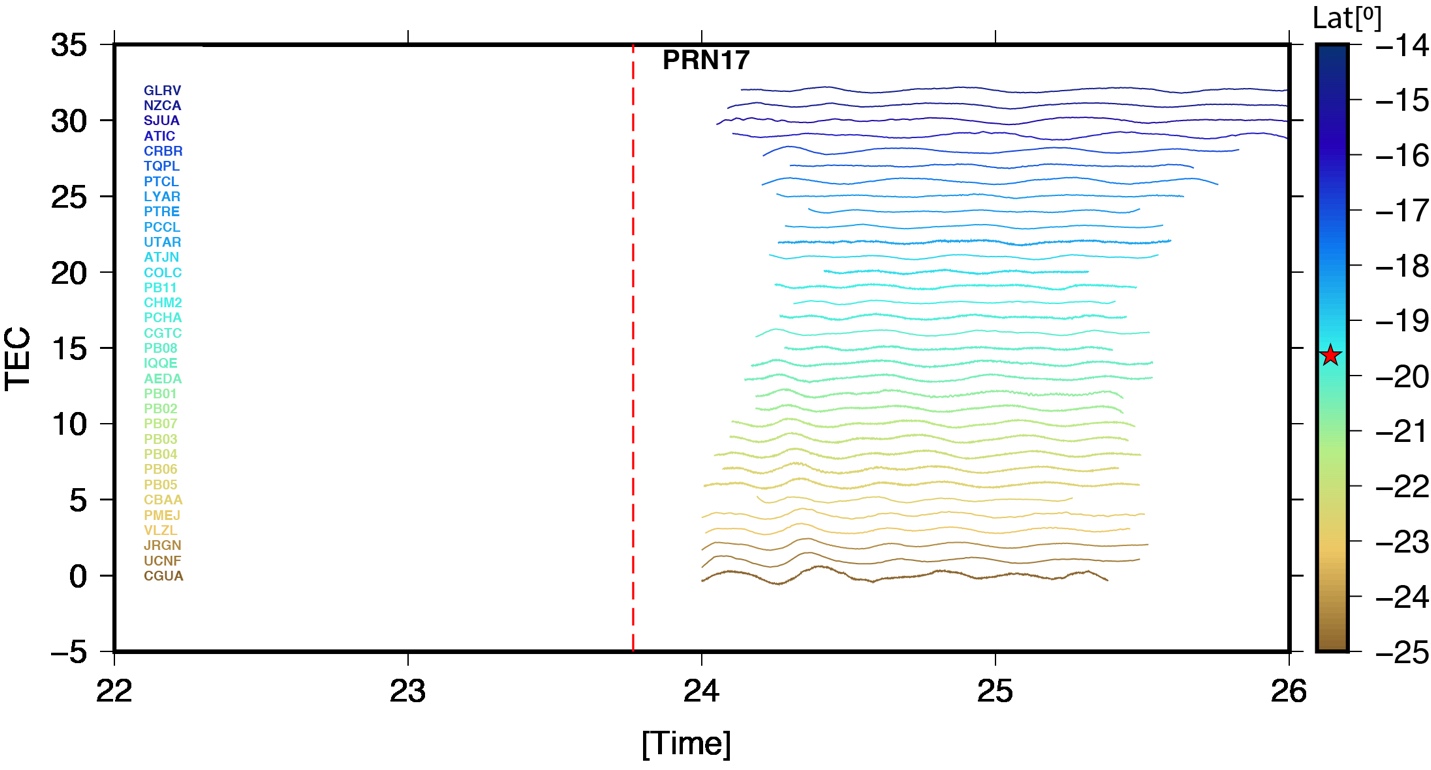


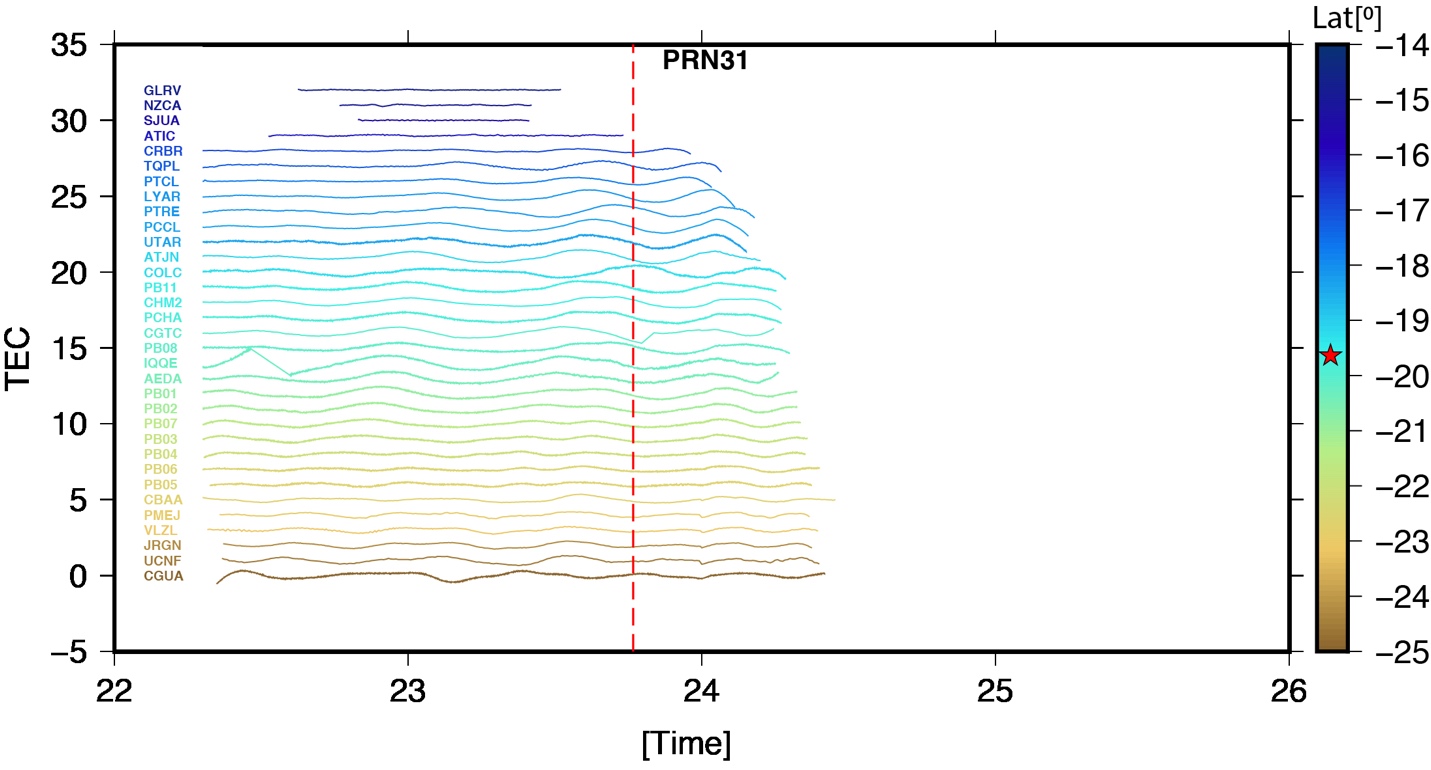


**Supplementary Figure S1.** VTEC of the Pisagua region of PRN 13, 17, 31 in the low elevation angle.


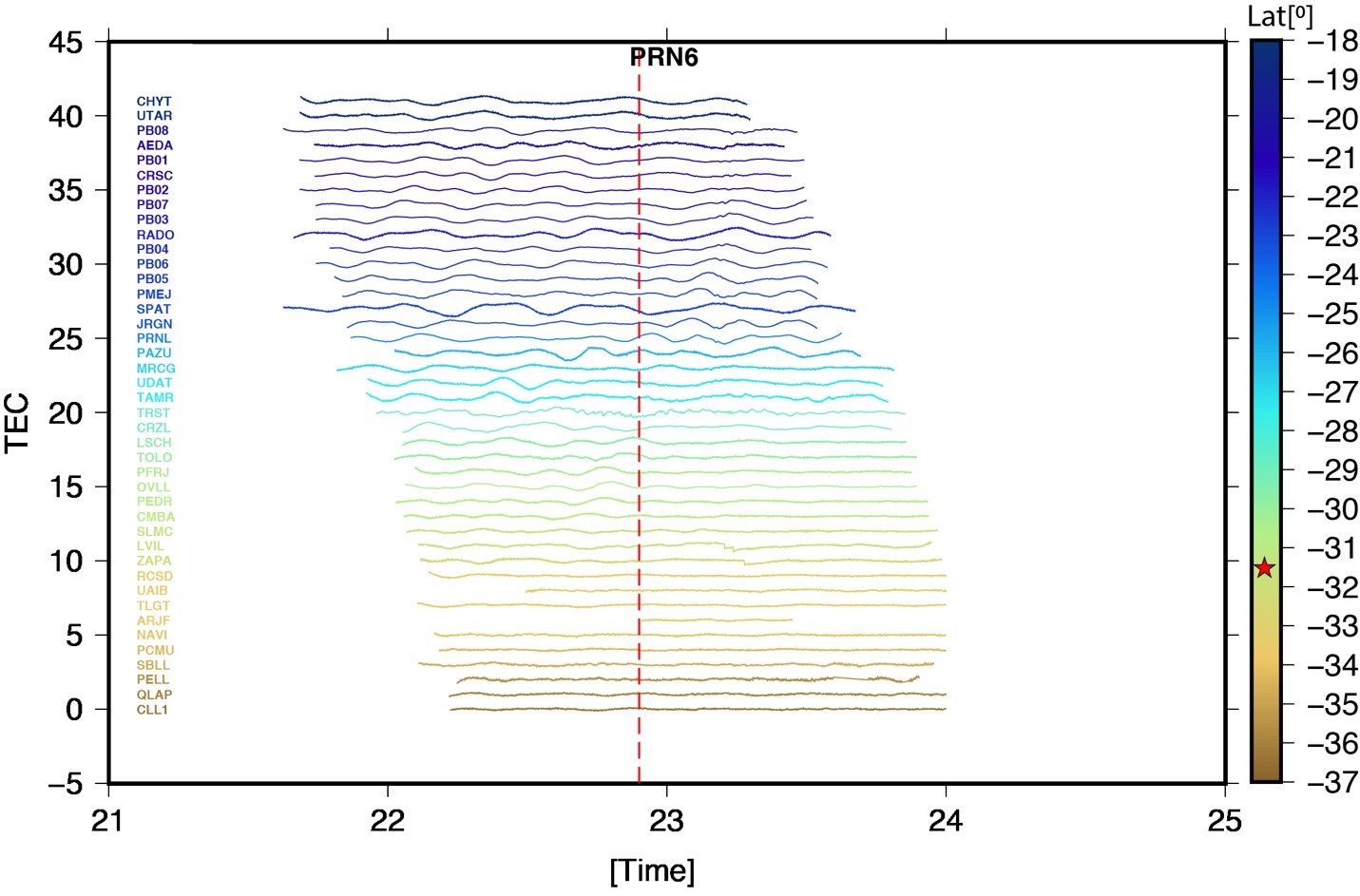


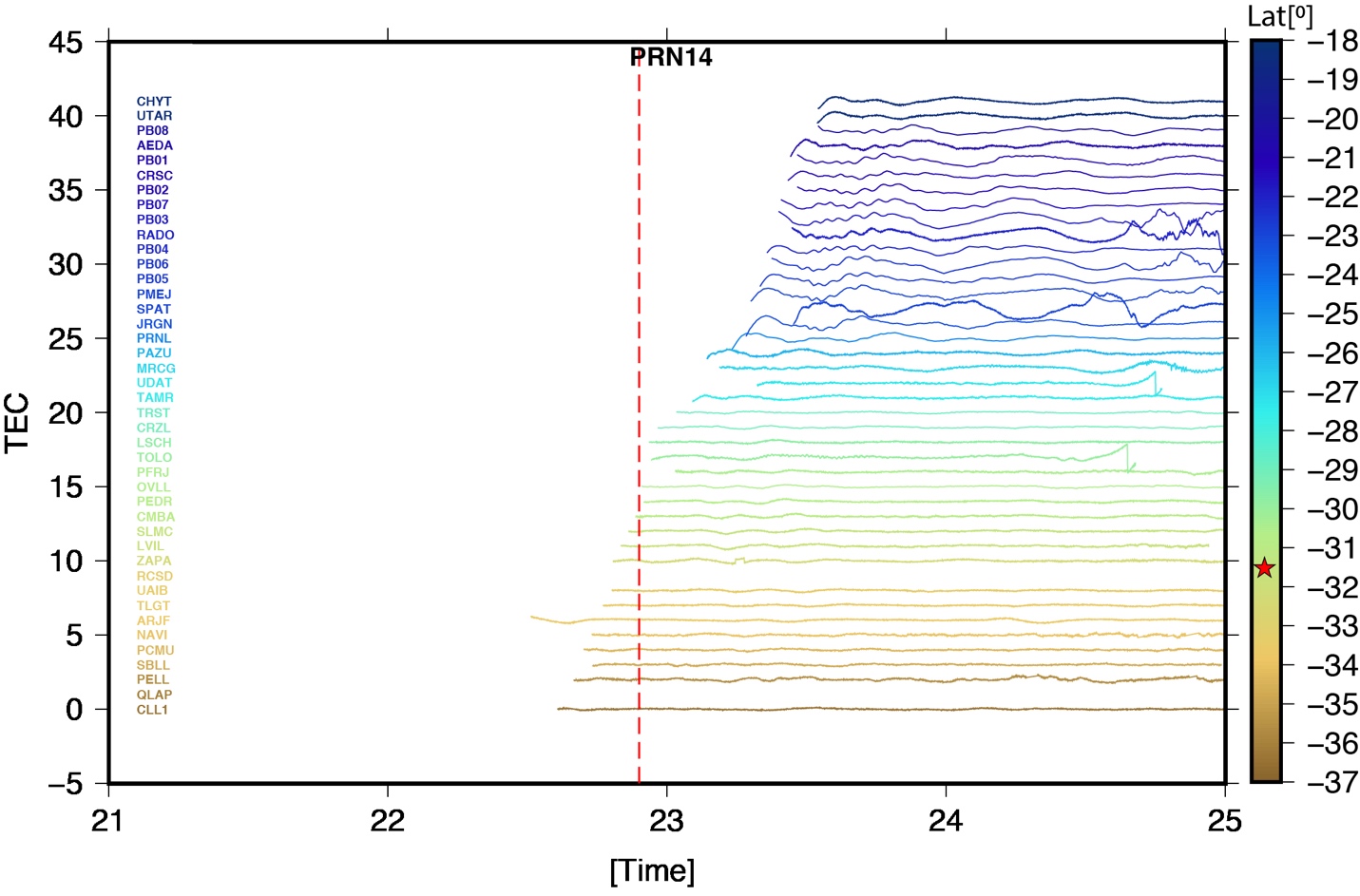


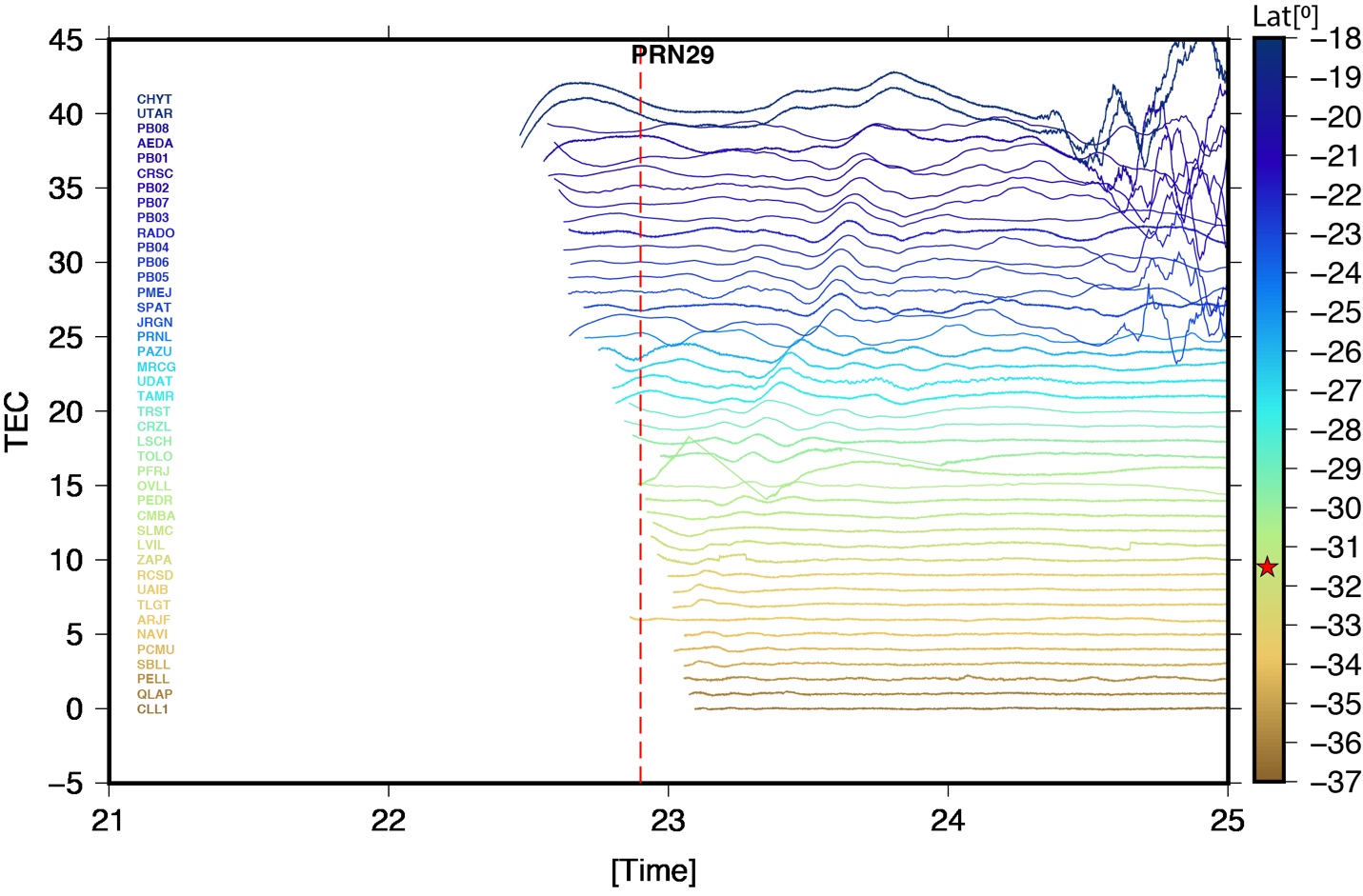


**Supplementary Figure S2.** VTEC of the Pisagua region of PRN 06, 14, 29 in the low elevation angle.


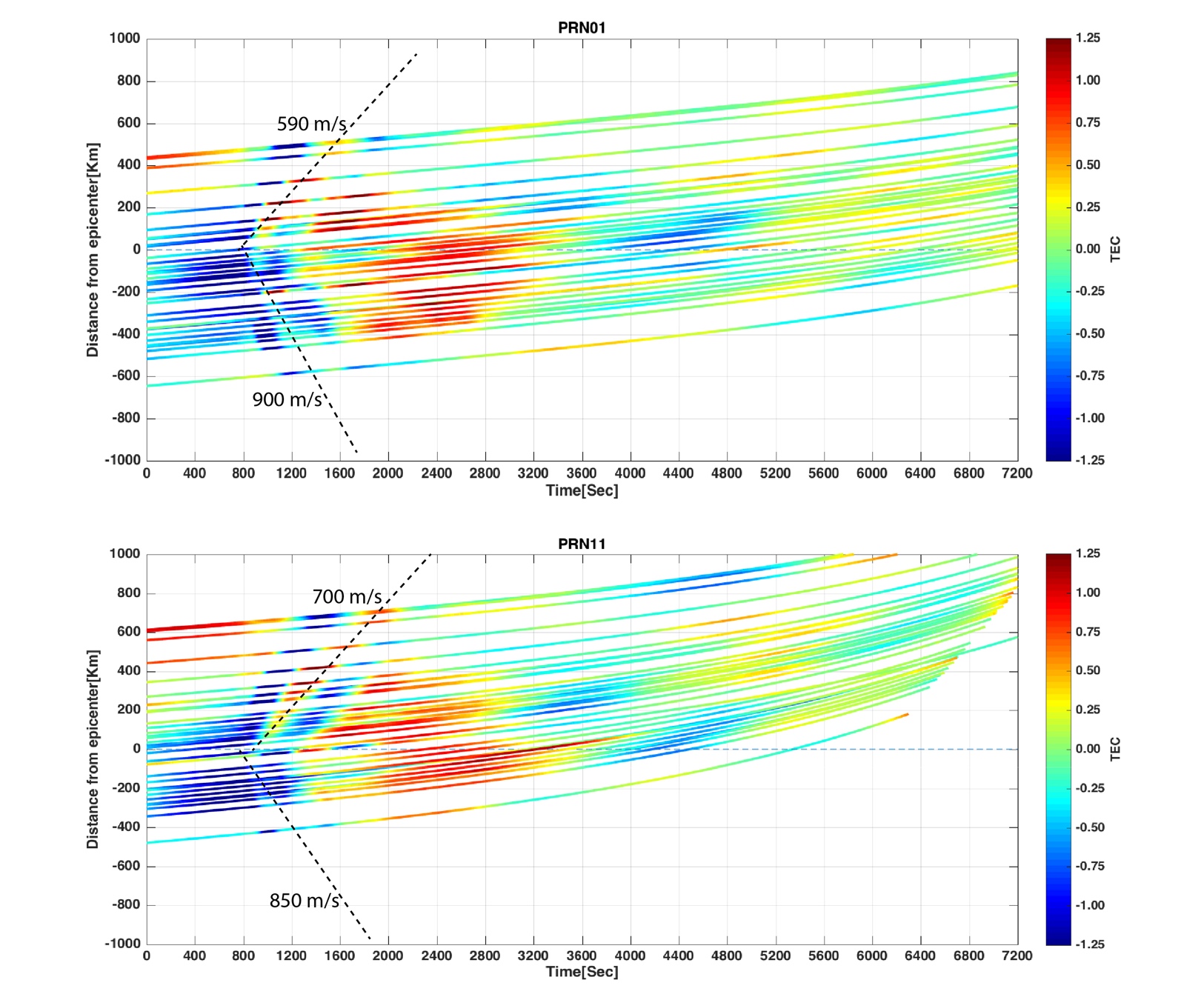


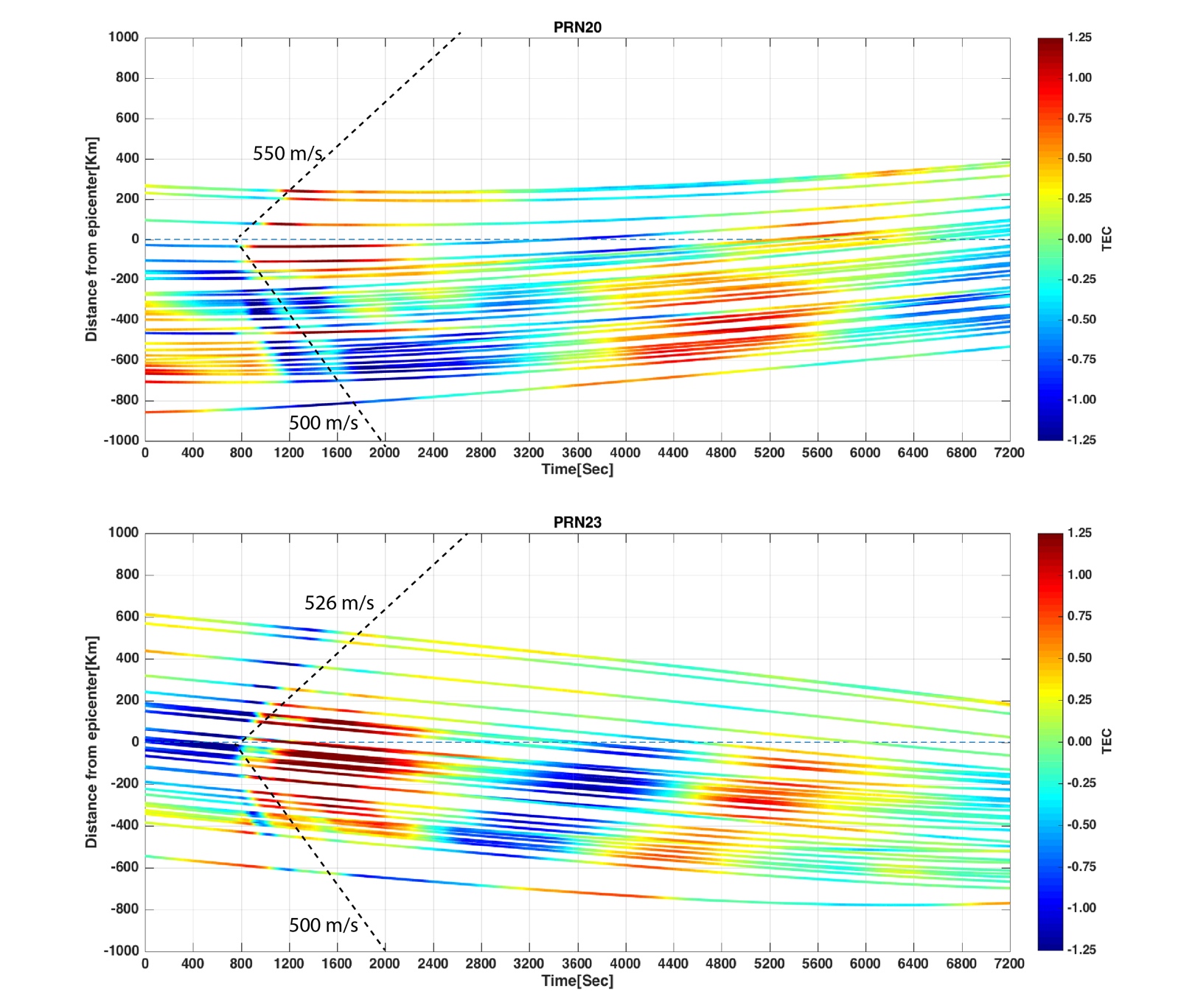


**Supplementary Figure S3:** Hodochrone map of Pisagua earthquake region of PRN 01,11,20 and 23.


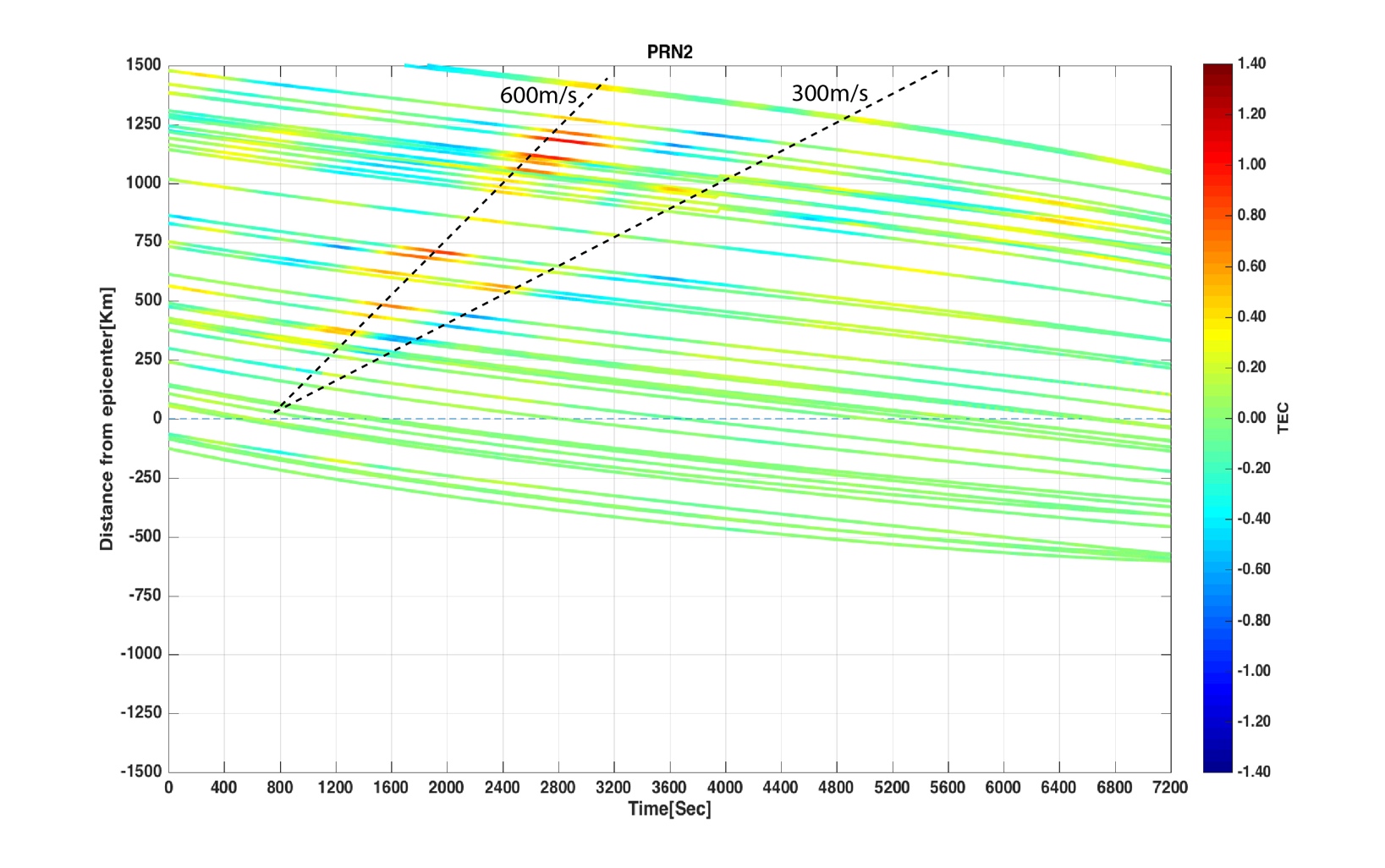


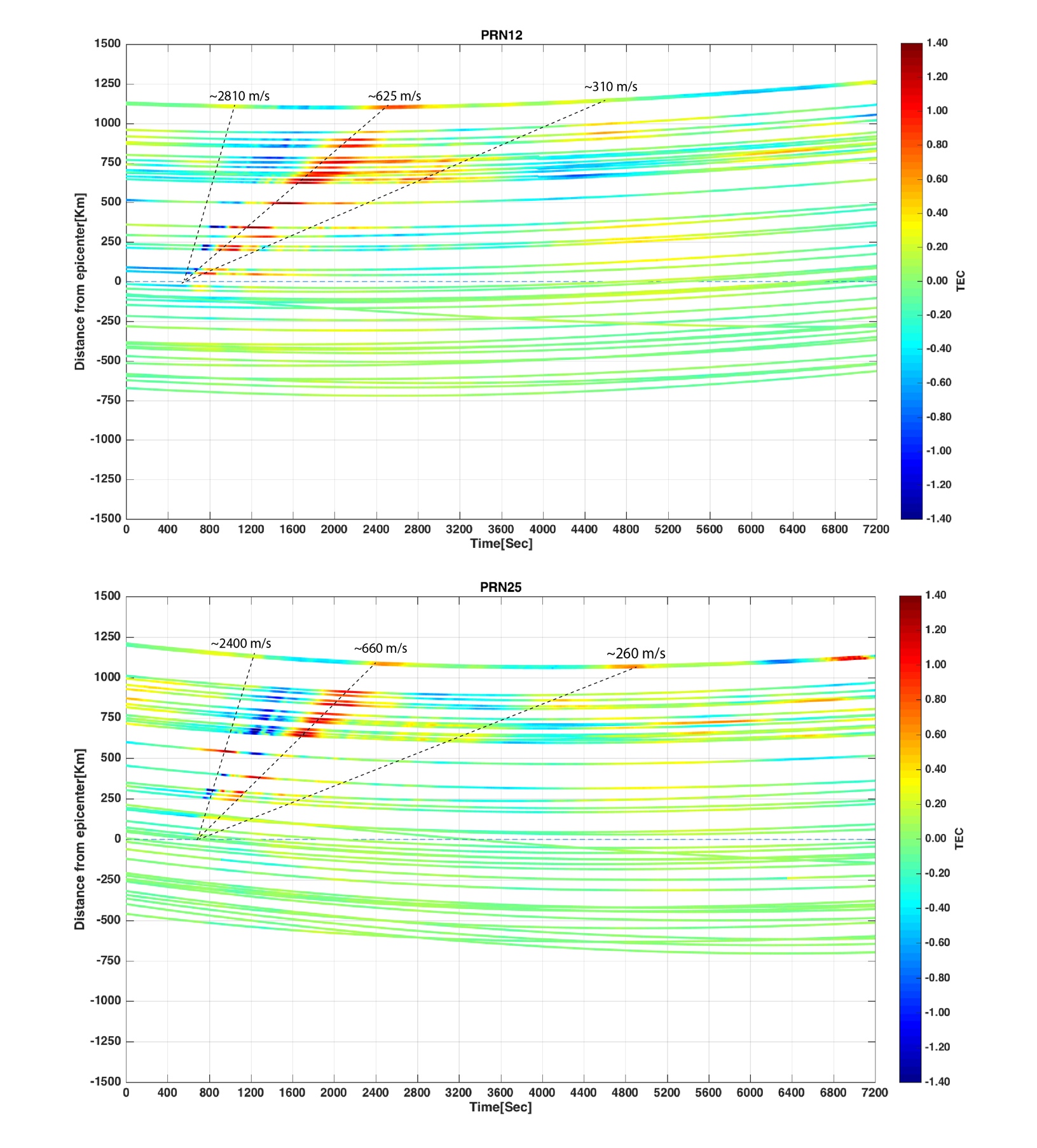


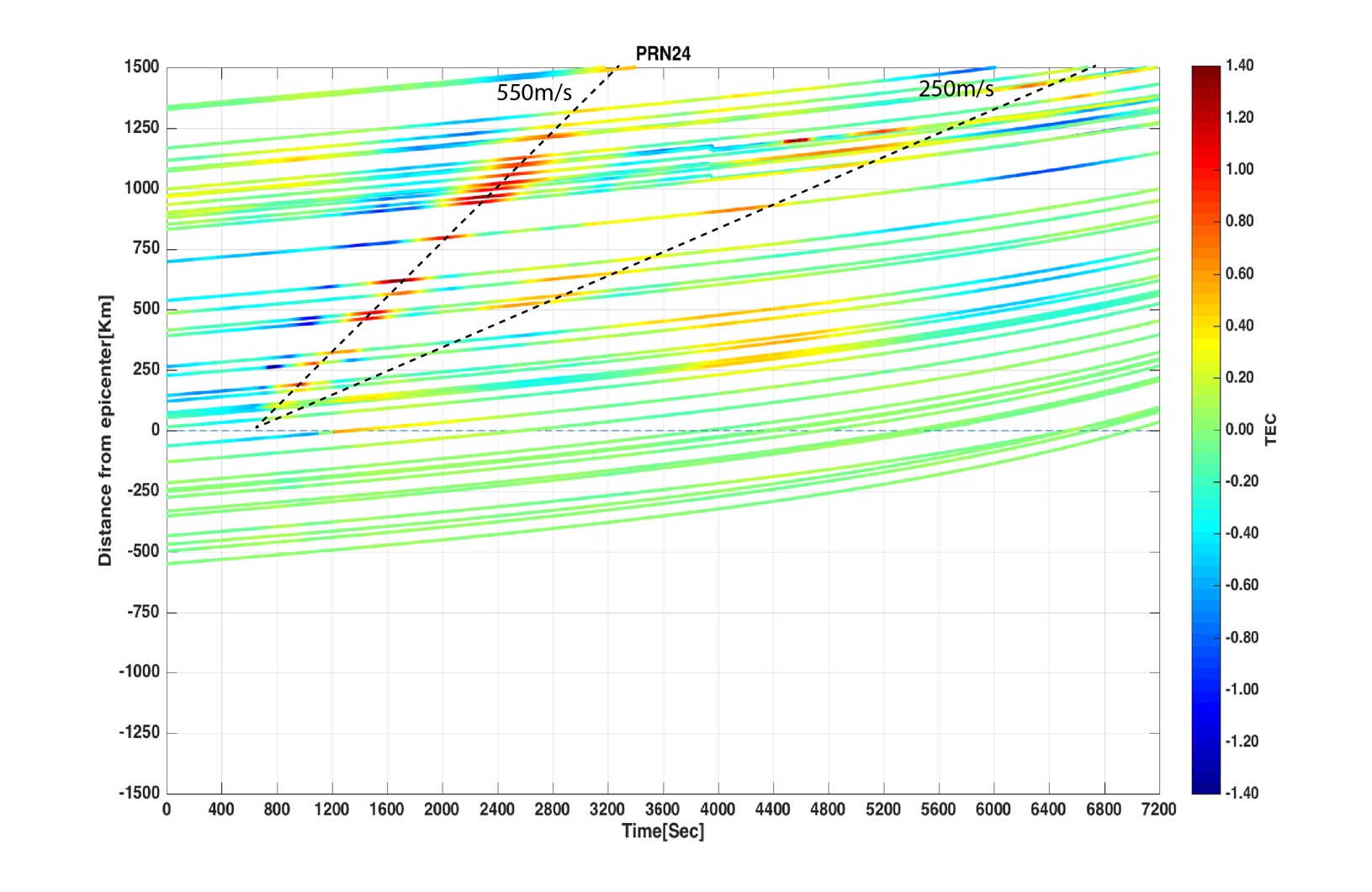


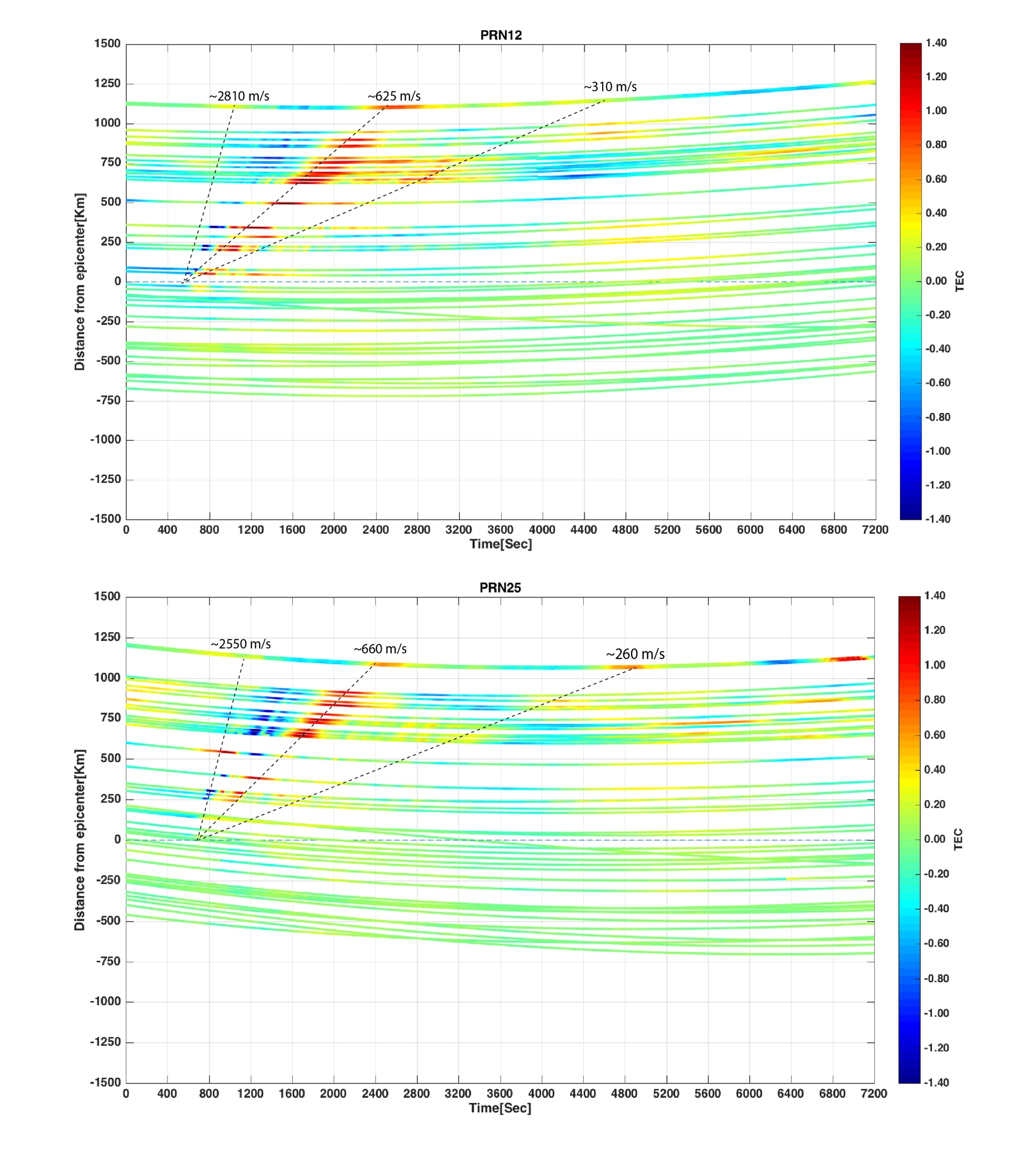


**Supplementary Figure S4:** Hodochrone map of Illapel earthquake region of PRN 02,12,24 and 25.
